# Bone marrow fibrosis as a feature of systemic lupus erythematosus: a case report and literature review

**DOI:** 10.1186/2193-1801-3-349

**Published:** 2014-07-09

**Authors:** Émilie Chalayer, Martine Ffrench, Pascal Cathébras

**Affiliations:** Service de médecine interne, Hôpital Nord, CHU de Saint-Étienne, Saint-Étienne, Cedex 2, 42055 France; Laboratoire d’hématologie, Centre Hospitalier Lyon Sud, 165 Chemin du Grand Revoyet, 69495 Pierre Bénite, France

**Keywords:** Primary myelofibrosis, Bone marrow, Systemic lupus erythematosus

## Abstract

**Introduction:**

Peripheral cytopenias are common in systemic lupus erythematosus (SLE), but bone marrow involvement is rarely reported. Myelofibrosis is a rare disorder characterized by reticulin fibrosis of the bone marrow, which usually occurs in response to clonal proliferation of hematopoietic stem cells in myeloproliferative disorders. However, bone marrow fibrosis has also been described in association with auto-immune diseases, especially SLE.

**Method:**

We will report here a new case of bone marrow fibrosis associated with SLE. We also reviewed the 27 cases published in the English language literature, and will discuss the clinical presentation, outcome, treatment, and pathophysiology of bone marrow fibrosis occurring in association with SLE.

**Results:**

Over one half of patients were diagnosed concomitantly with bone marrow fibrosis and SLE. Epidemiological, clinical and biological features of lupus were unremarkable. Except for the presence of reticulin fibrosis, the findings from the bone marrow biopsies proved highly variable. Overall mortality was about 14% but corticosteroid-based therapy lead to clinical improvement and reverted bone marrow fibrosis in most cases. Data on the usefulness of other immunomodulatory therapies are inconclusive.

**Conclusions:**

SLE may be complicated by bone marrow involvement, of a likely autoimmune origin. Bone marrow fibrosis occurring with SLE is probably similar to “primary autoimmune myelofibrosis” and may respond to steroid and immunomodulatory therapies. Further studies with standardised proofreading of bone marrow aspirations and biopsies are needed to delineate the clinical and biological features of this rare complication of SLE.

## Introduction

Hematological abnormalities such as anemia, auto-immune hemolysis, leukopenia, lymphopenia and thrombocytopenia are very common in systemic lupus erythematosus (SLE) (Beyan et al. 2007). Blood cytopenias are among the criteria for SLE in the revised American College of Rheumatology (ACR) (Hochberg 1997) guidelines and may occur as the first symptom of the disease. Most of these manifestations are caused by increased peripheral destruction of blood cells associated with circulating auto-antibodies. Occasional case reports and small series have documented bone marrow abnormalities in patients with SLE, such as myelofibrosis, aplastic anemia, pure red cell aplasia, and features suggestive of myelodysplastic syndromes, suggesting that the bone marrow may also be a target organ in the disease. Bone marrow fibrosis is defined by the deposition of reticulin fibres in the bone marrow stroma. Fibrosis usually occurs in response to the clonal proliferation of hematopoietic stem cells (Kuter et al. 2007). Sporadic reports have suggested that bone marrow fibrosis may be part of the SLE disease spectrum, and the relationship between disease control and pancytopenia improvement provides indirect evidence for a causal relationship between SLE and bone marrow fibrosis. Auto-immune myelofibrosis may respond to immunosuppressive treatment with regression of the fibrosis and regeneration of the normal marrow tissue (Pullarkat et al. 2003). However, bone marrow involvement in SLE has not been thoroughly studied and the etiological mechanisms of this rare complication remain unclear. We report here a case of bone marrow fibrosis associated with SLE. In order to improve the knowledge on this rare but serious complication of SLE, we have also reviewed all previously published cases.

## Case report

A 17 year-old woman with a history of polyarthralgias and photosentivity was referred to the hospital in 1999 for the evaluation of a Raynaud’s phenomenon. Clinical examination revealed no anomaly. She presented with neutropenia (leukocyte count 2.4 × 10^9^/l with 1.1 × 10^9^/l neutrophil granulocytes and 0.74 × 10^9^/l lymphocytes) without anemia or thrombocytopenia. Antinuclear antibodies were present (>1/1280 with a speckled fluorescence pattern). Anti-DNA antibodies were absent. Anti-U1-RNP and anti-SSA antibodies were positive. Three years later (2002), she was admitted to the hospital because of atypical eating disorder and psychotic behaviour. No evidence for neuropsychiatric lupus was found despite an extensive work-up. The patient was diagnosed with schizophrenia and treated with olanzapine. Nine years later (2011), she was referred to the hospital for fatigue, fever and pancytopenia. With the exception of extreme paleness, clinical examination was unremarkable. Her weight is normal. Laboratory findings on admission showed the following values: leukocyte count 0,48 × 10^9^/l with 0.28 × 10^9^/l neutrophil granulocytes and 0.08 × 10^9^/l lymphocytes, hemoglobin 2.9 g/dl, platelet count 15 × 10^9^/l and reticulocyte count 3 × 10^9^/l, low-normal level of folate, normal levels of vitamin B12 and ferritin. Anti-SSA, anti-RNP 70 and anti-U1-RNP antibodies were positive. The anti-DNA antibody titer was 76 UI/ml in ELISA [normal range < 10]. Direct Coombs’ test was negative. C3, C4, and CH50 were respectively 1.18 g/l [normal range: 0.79–1.52], 0.12 g/l [normal range: 0.2–0.51] and 120%. Abdominal ultrasonography showed limit homogen splenomegaly (around 12 cm). Tear drop cells or leukoerythroblastic blood smear were not noted. Two attempts to aspirate bone marrow at different sites were unsuccessful and yielded only dry taps. Bone marrow biopsy showed hypercellular marrow (cellularity: 80%) with focal lymphocytic infiltration, dysmyelopoiesis, erythrophagocytosis and grade 1–2 fibrosis. JAK-2, MPL W515L/K, and calreticulin mutations were not screen. A diagnosis of SLE with bone marrow involvement was retained. High dose methylprednisolone (500 mg/d) was given for 3 days. Then hydroxychloroquine and prednisone (1 mg/kg) were started, in addition to supportive care with blood transfusion and antibiotics. However, the patient remained pancytopenic. Intravenous immune globulins (30 g/d) were given for 4 days with remarkable improvement. Hemoglobin level after 2 months was 11.8 g/dl, platelet count was 175 × 10^9^/l and leucocyte count was 3.27 × 10^9^/l with 2.32 × 10^9^/l neutrophil granulocytes and 0.49 × 10^9^/l lymphocytes. Prednisone was continued for 17 months and gradually tapered. The patient did not experience change in her mental status during the course of steroid treatment. She remains in good physical health and still takes hydroxychloroquine and olanzapine.

## Review of published cases

### Method

An electronic search of the literature was performed via MEDLINE by crossing the key words “systemic lupus erythematosus” AND [“bone marrow fibrosis” OR “myelofibrosis”]. We then examined additional references from the retrieved articles. The study period ran from January 1975 through December 2013. Only papers written in English were reviewed.

### Results

#### Clinical presentation (Table 1)

**Table 1:** **Clinical presentation**

N ^ref^	Age (yrs)	M/F	PD SLE	SLE symptoms and signs	Associed symptoms and signs	LNE	HMG	SMG
1 (Sarkar et al. 2009)	45	M	-	Pleural effusion	Fever, melena, epistaxis	-	-	-
2 (Sacre et al. 2009)	44	F	+	Diffuse alopecia, arthritis				-
3 (Pillai et al. 2009)	40	F	-	Proteinuria, pleural and pericardial effusion, ascites	Fever	-	+	-
4 (Aziz et al. 2004)	22	F	-	Membranous nephropathy	Fever, epistaxis	+	+	+
5 (Kiss et al. 2000)	18	F	+	Alopecia, facial erythema, arthritis	Fever, weight loss, myositis		-	-
6 (Durupt et al. 2000)	29	F	+	Polyarthritis, mesangial glomerulonephritis	Fever, hematuria	-	-	-
7 (Vora et al. 1998)	22	F	+	Membranous nephropathy, seizures	Severe recurrent posterior scleritis			+
8 (Aharon et al. 1997; Amital et al. 2003)	54	F	-	Arthralgias, pleural effusion	Fever, weight loss, abdominal pain	+	+	+
9 (Agarwal et al. 1995)	12	F	+	NS	Fever, petechias	-	-	-
10 (Ramakrishna et al. 1995)	18	F	-	Alopecia, arthralgias, Evans’ syndrome	Fever, weight loss, menorrhagia, headaches	-	-	-
11 (Paquette et al. 1994)	68	M	-	Pleural effusion, oral ulcers				-
12 (Paquette et al. 1994)	27	F	-	NS	Epistaxis, hematuria, gingival bleeding, petechias	+		+
13 (Paquette et al. 1994)	23	F	+	Photosensitivity, alopecia, malar rash, pharyngeal ulcerations, arthritis, oral ulcers	Gingival bleeding, retinal lesion		+	+
14 (Paquette et al. 1994)	56	F	+	Alopecia, arthritis	Cutaneous vasculitis			-
15 (Paquette et al. 1994)	18	F	-	NS	Fever, menorrhagia, epistaxis, gingival bleeding, petechias	+	+	+
16 (Paquette et al. 1994)	70	F	-	Arthritis, pleural effusion	Fever, weight loss, hematemesis			-
17 (Paquette et al. 1994)	62	F	-	Psychosis	Fever, weight loss, panniculitis		+	+
18 (Paquette et al. 1994)	69	F	+	Arthritis, immune thrombocytopenia	Petechias		+	S
19 (Hirose et al. 1993)	54	F	+	Oral ulcers, polyarthralgias	Fever, weight loss			
20 (Borba et al. 1993)	39	F	+	Malar rash, photosentivity, alopecia, Raynaud’s, arthritis, pericarditis, pleural effusion	Fever			
21 (Foley-Nolan et al. 1992)	20	F	-	Arthralgias				-
22 (Inoue et al. 1992)	24	F	-	Proteinuria	Fever, purpura	-	-	-
23 (Matsouka et al. 1989)	60	F	-	Proteinuria, immune thrombocytopenia	Fever, weight loss, petechias and ecchymoses		+	S
24 (el Mouzan et al. 1988)	13	F	-	Arthritis	Fever, anorexia, petechias and ecchymoses	+	+	+
25 (Kaelin and Spivak 1986)	27	F	+	Hyperpigmented skin rash, polyarthralgias	Ecchymoses, epistaxis, melena, gingival bleeding, purpura	-	-	-
26 (Nanji and Jetha 1984)	28	M	+	Erythematous macular lesions, arthritis, deterioration in renal function, pleural and pericardial effusion, ascites	Fever		+	+
27 (Daly and Scott 1983)	16	F	-	Alopecia, arthritis	Weight loss, subcutaneous nodules in hands, retinal exudates, epistaxis, gingival bleeding, purpura and ecchymoses	+	+	+
Present	29	F	+	Photosensitivity, polyarthralgias and Raynaud’s phenomenon	Fever, edema	-	-	+

PD: previous diagnosis of SLE, LNE: lymph node enlargement, HMG: hepatomegaly, SMG: splenomegaly, S: splenectomy, NS: not specified.

Of the 27 cases retrieved from the English language literature and our case, 3 were males and 25 females, giving a ratio of 1:9. The age range was 12 to 70 years with a mean of 36 years and a median of 29 years. Fifteen patients received a concomitant diagnosis of SLE and bone marrow fibrosis, and 13 patients who had been previously diagnosed with SLE subsequently developed bone marrow fibrosis. In these 13 cases, the onset of bone marrow fibrosis varied from 8 months to 13 years after SLE diagnosis, with a mean of 5 years. Seven of these patients were under corticosteroids at the time of the hematological complication, two patients were under hydroxychloroquine, one had discontinued azathioprine at least 4 months before (Kiss et al. 2000) and one had started azathioprine two weeks before, but 4 weeks after discontinuing the drug no improvement had been observed (Vora et al. 1998). In patients with previously diagnosed SLE, symptoms attributed to the disease before bone marrow fibrosis occurred were rheumatologic symptoms (11/13), muco-cutaneous symptoms (8/13), renal involvement (3/13), serositis (3/13), and seizures (1/13). For all 28 patients, at the time of admission for cytopenias subsequently attributed to bone marrow fibrosis, the physical findings suggestive of SLE were rheumatologic symptoms (8/28), renal involvement (7/28), serositis (6/28) and muco-cutaneous symptoms (4/28). Splenomegaly was found in 11/26 patients (2 patients had undergone splenectomy due to immune thrombocytopenia), hepatomegaly in 11 patients, and lymph node enlargement or small diffuse lymph nodes in 6 patients. The other major symptoms reported were fever (17/28) and bleeding (15/28). Previous hematological history was often unclear, but at least 2 patients had been diagnosed with immune thrombocytopenia before the diagnosis of bone marrow fibrosis.

#### Peripheral hematological abnormalities (Table 2)

**Table 2:** **Biology**

N ^ref^	Age yrs	M/F	Hb g/dl	Pl 10 ^9^/l	WBC 10 ^9^/l	N 10 ^9^/l	L 10 ^9^/l	ANA	DNA	other	Co test	LC	Bone marrow examination
1 (Sarkar et al. 2009)	45	M	5.3	25	2.5	1.2	1	1/640	+		+		Hypercellular marrow with focal lymphocytic infiltration.
2 (Sacre et al. 2009)	44	F	7	65				high	+		-		Hypercellularity with marked reticulin fibrosis.
3 (Pillai et al. 2009)	40	F	10.6	25		0		1/320	-		+	+	Hypercellular with increased megakaryocytes and reticulin (grade 3).
4 (Aziz et al. 2004)	22	F	6.2	18	1.2			1/160				+	Hypercellularity with an increased number of megakaryocytes. Silver stain showed increased reticulin fibrosis and the presence of patchy areas of collagenized marrow.
5 (Kiss et al. 2000)	18	F	4.2	28	0.9			high		aCL			Significant increase in the amount of reticulin fibres (40%, Beumaister 3–4), hypocellularity of the myeloid components and massive lymphocytic infiltration.
6 (Durupt et al. 2000)	29	F	9.4	102	1.1	0.6	0.3	1/1120	+			+	All normal hematopoietic elements with a small increase in mature megakaryocytes and marked inflammatory medullar reaction with plasmocytosis, systematized increase in reticulin (grade 1).
7 (Vora et al. 1998)	22	F	9	60	4			1/1000	-				Marked osteomyelosclerosis with severe fibrosis of the marrow spaces and near-total effacement of normal hematopoiesis.
8 (Aharon et al. 1997; Amital et al. 2003)	54	F	5.1	96	3.1	2.2	0.6	high	+	aCL, anti-histone, anti-SSA		+	Focal hypercellularity, many reticulin and collagen fibers, normal appearance of the red cell and white cell lines, mild megakaryocytosis with few mildly abnormal megakaryocytes.
9 (Agarwal et al. 1995)	12	F	9.6	4	4.7	0.7	3.3	1/40	-		-		Extremely hypocellular marrow with residual patches of hematopoietic cells. Reticulin stain showed a marked increase in fine fibrosis, which was diffuse grade 3.
10 (Ramakrishna et al. 1995)	18	F	5.4	30	6			1/2560	+	LA, aCL, antiplatelet	+	+	Markedly hypercellular marrow with erythroid hyperplasia, plentiful megakaryocytes and markedly increased reticulin.
11 (Paquette et al. 1994)	68	M	5.7	222	3.5			1/5280	-			+	Hypercellular, increased stroma and reticulin fibrosis.
12 (Paquette et al. 1994)	27	F	5	1	5.9			1/80	-		+	-	Fibrosis with megakaryocyte clustering.
13 (Paquette et al. 1994)	23	F	9.5	55	4.2			high				-	Fibrosis, open sinusoids, megakaryocyte clusters.
14 (Paquette et al. 1994)	56	F	9.7	76	5.4			high	+		-	+	Erythroid hyperplasia, increased megakaryocytes, fibroblast proliferation, reticulin fibrosis.
15 (Paquette et al. 1994)	18	F	2.7	4	7.7			high	+		+	+	Bone marrow fibrosis, hypercellular marrow, increased megakaryocytes with clustering.
16 (Paquette et al. 1994)	70	F	4.2	20	3.8			high	-		+	+	Advanced bone marrow fibrosis, hypocellular marrow, predominance of megakaryocytes.
17 (Paquette et al. 1994)	62	F	9.3	35	3			high	-			+	Advanced bone marrow fibrosis, marked osteosclerosis.
18 (Paquette et al. 1994)	69	F	6.8	39	6.8			high	-			-	Hypercellular with increased, clustering megakaryocytes; stroma moderately increased by accumulation of fibrillar reticulin.
19 (Hirose et al. 1993)	54	F	6.9	17	1.7			1/320	-	aCL, LA		+	Marked marrow fibrosis with reduced numbers of erythroid precursors and normal proliferation of both megakaryocytes and myeloid series, the reticulin content was increased.
20 (Borba et al. 1993)	39	F	9.7	341	2.4	0.9		1/200	-	anti-neutrophil	+	+	All normal hematopoeitic elements with localised fibrosis, reticulin was moderately increased.
21 (Foley-Nolan et al. 1992)	20	F	10.5	45	4.5	1.3	1.9	1/800			+	+	Hypercellular with a marked increase in the number of megakaryocytes and a diffuse, significant increase in reticulin content.
22 (Inoue et al. 1992)	24	F	6.8	2	3.8			1/100	-	antiplatelet	-	+	Bone marrow fibrosis characterized by marked hyperplasic marrow with an increase in reticulin fibers, all elements including megakaryocytes increased in number.
23 (Matsouka et al. 1989)	60	F		20	4	1.4	1.4	1/320			-	-	Presence of dense fibrous tissue with fibroblasts, paucity of immature and polymorphonuclear cells.
24 (el Mouzan et al. 1988)	13	F	6.5	10	1.3	0.2	1	1/640	+	rhumatoid factor	+	+	Decreased bone marrow activity with 1:1 myeloid to erythroid ratio, megakaryocytes quantitatively normal, increased reticulo-endothelial activity and fibrosis.
25 (Kaelin and Spivak 1986)	27	F	13.1	5	4.4	2.8	0.9	1/1280	+	antiplatelet, anti-neutrophil	+	+	Contained all normal hematopoietic elements with a slight increase in mature megakaryocytes, markedly increased reticulin.
26 (Nanji and Jetha 1984)	28	M	8.4	1	0.3			high			-		Hypocellular in some areas and hypercellular in others, megakaryocytes decreased in number, increased numbers of histiocytes and fibroblasts, extensive fibrosis and diffuse increased in reticulin.
27 (Daly and Scott 1983)	16	F	7	28	1.7	0.5		1/1280	+		-	+	Marked marrow fibrosis with reduced numbers of erythroid precursors, plentiful megakaryocytes, greatly increased reticulin content.
Present	29	F	2.9	15	0.48	0.28	0.08	1/1280	+	anti-SSA, RNP 70, U1-RNP	-	+	Hypercellular marrow (cellularity : 80%) with focal lymphocytic infiltration, dysmyelopoiesis, erythrophagocytosis and grade 1–2 fibrosis.

Hb: hemoglobin, Pl: platelets, WBC: white blood count, N: neutrophils, L: lymphocytes, ANA: anti-nuclear antibodies, DNA: anti double-stranded DNA antibodies, Co test: Coombs test, LC: low complement, LA: lupus anticoagulant, aCL: anticardiolipin.

Thirteen of the 28 patients had pancytopenia (anemia: Hb <10 g/dl, leukopenia: WBC < 4 × 10^9^/l, and thrombocytopenia: platelets < 150 × 10^9^/l), 13/28 had bicytopenia (anemia or/and leukopenia or/and thrombocytopenia), 1/28 had thrombocytopenia and neutropenia without leukopenia and 1/28, only thrombocytopenia. Neutropenia (<1,5 × 10^9^/l) was observed in 10/12 (16 missing data) patients, lymphopenia in 4/9 (19 missing data), hemolysis with hyper-reticulocytosis in one case and a positive direct Coombs’ test in 10/18 (10 missing data). Hemoglobin levels ranged from 13.1 to 2.7 g/dl (mean 7.3 g/dl), platelets from 341 to 1 × 10^9^/l (mean 50 × 10^9^/l), and leucocytes from 6.8 to 0.35 × 10^9^/l (mean 3.4 × 10^9^/l). Of the 24 patients with thrombocytopenia, 11 had deep (<20 × 10^9^/l), 8 severe (<50 × 10^9^/l), and 5 moderate thrombocytopenia. Tear drop cells or leukoerythroblastic blood smear, two common finding in primary myelofibrosis, were noted in 14 patients.

#### Bone marrow abnormalities (Table 2)

For 22/28 patients, a “dry tap” occurred during bone marrow aspiration. All bone marrow biopsies showed bone marrow fibrosis with variable increases in the amount of reticulin fibers and fibroblasts. Grades of bone marrow fibrosis were not always specified. Global marrow cellularity was variable, ranging from increased (12/28) to normal (11/28) or decreased (5/28) without any increase in blasts. All elements including megakaryocytes appeared morphologically normal. Megakaryocytes were increased or/and with clustering in 13/28 patients and decreased in 1/28. Focal or massive lymphocytic infiltration was observed in 4/28, plasmocytosis in 1/28 and erythroid hyperplasia in 2/28. Fifteen patients underwent repeated bone marrow examination showing improvement, with reduction in reticulin amounts in 12/15.

#### Immunological abnormalities (Table 2)

Antinuclear antibodies were found in all patients, anti-dsDNA in 12/28, anti-Ro/SSA in 2/28, anti-histone in 1/28, and a low complement level in 18/28 patients. Antiphospholipid antibodies were detected in 4/28 patients. Anti-platelet antibodies testing came out positive in 3 patients and negative in 4.

#### Outcome and treatment (Table 3)

**Table 3:** **Treatment**

N ^ref^	Age (yrs)	Sex (M/F)	Medication received before bone marrow fibrosis	Immunomodulatory therapy for bone marrow fibrosis	Response
1 (Sarkar et al. 2009)	45	M		Prednisolone 60 mg/d	Improved
2 (Sacre et al. 2009)	44	F	Prednisone, hydroxychloroquine	Prednisone 1 mg/kg/d, IVIg	Improved
3 (Pillai et al. 2009)	40	F		IV methylprednisolone 500 mg/d, prednisone	Improved
4 (Aziz et al. 2004)	22	F		Prednisone 1 mg/kg/d	Improved
5 (Kiss et al. 2000)	18	F	Prednisone, azathioprine discontinued 6 months prior	IV methylprednisolone 1 g/d ×3, prednisone 2 mg/kg/d, cyclosporine 3 mg/kg, azathioprine	Relapsed, secondary improvement
6 (Durupt et al. 2000)	29	F		Prednisone 2 mg/kg/d	Improved
7 (Vora et al. 1998)	22	F	Prednisone, azathioprine initiated 2 weeks prior	Azathioprine discontinuation, 6 plasma exchanges, IV methylprednisolone 1 g pulses	Improved
8 (Aharon et al. 1997; Amital et al. 2003)	54	F		Prednisone 80 mg/d, IVIg 400 mg/kg/d ×5	Improved
9 (Agarwal et al. 1995)	12	F		Prednisone 2 mg/kg/d	Improved
10 (Ramakrishna et al. 1995)	18	F		Prednisolone 75 mg/d, IVIg, splenectomy, danazol, colchicine, vincristine	Relapsed, secondary improvement
11 (Paquette et al. 1994)	68	M		Prednisone 20 mg/d	Not improved
12 (Paquette et al. 1994)	27	F		Prednisone 60 mg/d	Improved
13 (Paquette et al. 1994)	23	F		Prednisone 50 mg/d	Improved
14 (Paquette et al. 1994)	56	F	Prednisone	Prednisone	Not improved
15 (Paquette et al. 1994)	18	F		Prednisone 80 mg/d	Improved
16 (Paquette et al. 1994)	70	F		Prednisone	Deceased
17 (Paquette et al. 1994)	62	F			Deceased
18 (Paquette et al. 1994)	69	F		Prednisone, splenectomy	Relapsed, secondary improvement
19 (Hirose et al. 1993)	54	F		IV methylprednisolone 1 g/d ×3, prednisone 60 mg/d	Improved
20 (Borba et al. 1993)	39	F	Prednisone, hydroxychloroquine	Methylprednisolone, prednisone, plasma exchanges, cyclophosphamide	Relapsed, secondary Improvement
21 (Foley-Nolan et al. 1992)	20	F		Prednisolone 40 mg/d, azathioprine 50 mg/d	Relapsed, secondary improvement
22 (Inoue et al. 1992)	24	F		Prednisolone 1,2 mg/kg/d, IV methylprednisolone 1 g/d ×3	Improved
23 (Matsouka et al. 1989)	60	F		Hydrocortisone 1 g/d	Deceased
24 (el Mouzan et al. 1988)	13	F		Prednisolone 30 mg/d	Improved
25 (Kaelin and Spivak 1986)	27	F	Prednisone, salicyclate	IV methylprednisolone 100 mg/d ×6, prednisone 50 mg/d	Improved
26 (Nanji and Jetha 1984)	28	M	Prednisone		Deceased
27 (Daly and Scott 1983)	16	F	NSAID including oxyphenbutazone	Prednisolone 30 mg/d	Improved
Present	29	F	Olanzapine	IV methylprednisolone 500 mg/d ×3, prednisone 1 mg/kg and hydroxychloroquine, IVIg 30 g/d ×4	Improved

NSAID: non-steroidal anti-inflammatory drugs. IVIg: intravenous immunoglobulins.

Follow-up time ranged from a few months to years but was often unspecified. The overall mortality was 14% (4/28). Two patients died within few days with no other treatment than blood transfusions and antibiotics. Two other patients who died received only prednisone as a specific treatment. Improvement was noted in 17/28 patients, transient response with need for new treatment in 5/28, and no improvement in 2/28. Supportive care with antibiotics and transfusions was explicitly mentioned for 8 patients. Two patients received granulocyte colony stimulating factor (G-CSF).

Immunomodulatory therapies consisted in corticosteroids (26/28) (prednisone, prednisolone or methylprednisolone), intravenous immune globulins (4/28) (Ramakrishna et al. 1995; Aharon et al. 1997; Sacre et al. 2009), plasma exchanges (2/28) (Borba et al. 1993; Vora et al. 1998), azathioprine (2/28) (Foley-Nolan et al. 1992; Kiss et al. 2000), cyclophosphamide (1/28) (Borba et al. 1993), cyclosporine (1/28) (Kiss et al. 2000), danazol (1/28) (Ramakrishna et al. 1995), colchicine (1/28) (Ramakrishna et al. 1995), vincristine (1/28) (Ramakrishna et al. 1995) and splenectomy (1/28) (Ramakrishna et al. 1995). Of 20 patients who received only corticosteroids, 16 improved and 4 did not. Four patients who received cortisone concomitantly with azathioprine, intravenous immunoglobulin or cyclosporine improved. One patient received cortisone and showed a transient response but pancytopenia relapsed so she received danazol, vincristine, colchicine, intravenous immunoglobulins, then underwent splenectomy and finally improved (Ramakrishna et al. 1995). Another patient was treated with plasma exchanges and cyclophosphamide following a transient response to cortisone and improved (Borba et al. 1993). One patient received intravenous immune globulins after 3 weeks of corticosteroid treatment without response, and a marked improvement occurred within the following week (Aharon et al. 1997).

### Discussion

#### Nosology

Primary myelofibrosis is considered as a clonal myeloproliferative disorder (Tefferi et al. 2012). However some diseases such as infections, neoplasms and autoimmune diseases may also induce bone marrow fibrosis. The term “myelofibrosis” is used in some contexts to describe any increase in bone marrow stromal fibres, regardless of the associated disease, and in other contexts to define a specific myeloid disorder (primary myelofibrosis) (Kuter et al. 2007). The word “myelofibrosis” is therefore ambiguous, and in this article we have chosen rather to use the term “bone marrow fibrosis”. Some authors suggest the importance of distinguishing between increases in bone marrow reticulin and collagen. Above-normal reticulin amounts are generally regarded as a nonspecific sign of bone marrow abnormality, but may or may not be a sign of serious neoplastic disease. In contrast, increased collagen is less common and is mainly seen in tumours metastatic to the bone marrow or in the late stages of myeloproliferative diseases. Unlike increased reticulin, it is not always reversible (Kuter et al. 2007). In most cases reported here, it was unclear if trichrome collagen stain and/or reticulin stain were performed, and the type and amount of fibrosis were not reported according to established grading scales (Kuter et al. 2007). Another issue is whether finding bone marrow reticulin fibrosis *per se* should prompt a diagnosis of autoimmune myelofibrosis in a patient with SLE. For example, mild degrees of reticulin fibrosis can be observed in conditions such as immune thrombocytopenia and may be found in many patients with lupus when routine bone marrow biopsies are performed (Pereira et al. 1998). Moreover, some authors have reported cases of bone marrow fibrosis in patients who do not have SLE or other well-defined autoimmune syndromes (Bass et al. 2001; Pullarkat et al. 2003). They have defined “primary autoimmune myelofibrosis” as a disorder characterized by cytopenias with bone marrow lymphocyte infiltration and grade 3 - 4 reticulin fibrosis of the bone marrow, lack of atypical bone marrow cells or osteosclerosis, absent or mild splenomegaly, and the presence of auto-antibodies. In our review, the 28 retrieved cases have been considered as fulfilling criteria for SLE, although lupus symptoms and signs leading to the diagnosis of SLE were not always reported in detail by the authors. Their clinico-pathological features were very similar to those of the reported cases of “primary autoimmune myelofibrosis”. Thus we tend to believe that “autoimmune myelofibrosis”, just like autoimmune cytopenias, may occur as an isolated disorder, or as a feature of other autoimmune diseases including SLE. Finally, cases of aplastic anemia have also been reported in SLE patients. We found 25 published cases in the English language literature (Aplastic anemia as a feature of systemic lupus erythematosus. In preparation). In these cases, the bone marrow biopsy showed marked hypocellularity, but the absence of reticulin fibrosis was often not specified, and thus the differentiation between “lupus bone marrow fibrosis” and “lupus aplastic anemia” is not always clear, raising the question of the borderland between these two rare features of SLE (Cavalcant et al. 1978).

#### Pathophysiology

The pathogenesis of bone marrow fibrosis remains incompletely understood, but appears to be a relatively nonspecific response of fibroblasts to underlying cellular abnormalities. Increased reticulin is the result of fibroblast proliferation, and increased collagen synthesis or altered collagen turnover appear to be due to decreased collagenase release from macrophages and neutrophils (Kuter et al. 2007). Several growth factors appear to be implicated. The platelet-derived growth factor (PDGF), found in megakaryocytes and platelets, stimulates fibroblast growth (Kuter et al. 2007). The transforming growth factor β (TGFβ) and epidermal growth factor (EGF) are known to promote collagen synthesis (Le Bousse-Kerdilès et al. 2008). Immunological abnormalities may be involved in the pathogenesis. The increased circulating immune complexes and auto-antibodies that are present in SLE may act on megakaryocyte Fc receptors and release growth factors to promote marrow fibrosis. Some authors have suggested that both auto-antibodies against CD34+ stem cells and cytotoxic T cells may initiate and perpetuate damage to the bone marrow (Kiss et al. 2000). An increase in leucocyte apoptosis and impaired clearance of apoptotic cells has also been observed in patients with SLE. These apoptotic bodies were observed in the bone marrow of patients with SLE, while they are not typically seen in normal bone marrow. Delayed apoptotic cell clearance leads to prolonged exposure of auto-antigens and predisposes to antibody production (Hepburn et al. 2007). Furthermore, in the bone marrow of patients with bone marrow fibrosis and SLE, megakaryocyte counts are often above normal or normal. Therefore thrombocytopenia may result at least partly from an increased destruction of the platelets rather than a decreased production caused by bone marrow fibrosis. An association between immune thrombocytopenia (to which bone marrow dysfunction is increasingly believed to contribute (Gernsheimer 2009)) and bone marrow fibrosis has been observed in 3 of the 28 reported cases.

The JAK2 V617F mutation, associated with primary myeloproliferative disorders, is present in up to one half of the patients with primary myelofibrosis (Tefferi et al. 2012). Some authors suggest a thorough search for auto-immunity in the absence of the mutation (Sacre et al. 2009).

#### Clinical and biological presentation

Primary myelofibrosis is diagnosed relatively late in life (median age is 66 years) and is more common in males (ratio 3:2) (Tefferi et al. 2012). Bone marrow fibrosis occurring with SLE is diagnosed earlier (median age is 29 years) and is very uncommon in males (ratio 1:9). In 15/28 cases, the diagnosis of SLE and bone marrow fibrosis were made simultaneously. However, in 5 of these cases a number of symptoms and signs (such as arthralgias, alopecia, proteinuria) were suggestive of undiagnosed yet pre-existing SLE (Daly and Scott 1983; Matsouka et al. 1989; Inoue et al. 1992; Paquette et al. 1994; Pillai et al. 2009). Some authors suggest that autoimmune disorders, including SLE, may be considered in cases of bone marrow fibrosis in patients whose spleen is not enlarged (Pullarkat et al. 2003; Sacre et al. 2009), but 10/26 patients in our review had splenomegaly. Moreover, in primary myelofibrosis, the clinical finding of splenomegaly is associated with collagen, but not reticulin fibrosis (Thiele and Kvasnicka 2006). Fifteen patients underwent repeated bone marrow examinations showing improvement, with a reduction in reticulin in 12/15. This suggests that reticulin fibrosis (and maybe even collagen fibrosis) can be reversed if the underlying disease is treated (Pereira et al. 1998).

#### Outcome and treatment

Although it is likely that negative outcomes are less frequently reported in case reports, for which follow up data may be lacking, and that consequently the overall mortality may be higher than the 14% documented from this review, this mortality rate suggests a more favourable course for SLE-associated bone marrow fibrosis than for primary myelofibrosis (Tefferi et al. 2012). Interestingly, bone marrow fibrosis occurring with SLE appears to often respond to corticosteroids, unlike primary myelofibrosis. Plasma exchanges seem to have no efficacy. Intravenous immune globulins were used for 4 patients only (Ramakrishna et al. 1995; Aharon et al. 1997; Sacre et al. 2009), and proved to be efficient in at least one patient (Aharon et al. 1997), as it was the case for the patient we managed. None of the 28 patients received rituximab. None received an allogeneic hematopoietic stem cell transplant.

### Conclusion

SLE may be complicated by bone marrow fibrosis, which is likely to be of autoimmune origin. This feature may be more common than previously thought, with cases being incorrectly characterized as blood peripheral cytopenias in patients previously diagnosed with SLE, and cases being misdiagnosed with primary myelofibrosis in patients not previously diagnosed with SLE. We think that in patients with SLE, cytopenias should be confirmed by bone marrow aspiration, and by bone marrow biopsy in atypical or refractory cases. Moreover, autoimmune myelofibrosis or SLE-associated bone marrow fibrosis should be considered in cases of primary myelofibrosis with atypical features such as young age and female sex, absence of spleen enlargement, or absence of JAK2 V617F mutation, because this condition seems amenable to efficient treatment. High-dose corticosteroid therapy with or without intravenous immune globulins should be the first-line therapy.

In order to improve knowledge of bone marrow involvement in SLE, we have established a French registry, with centralized proofreading of bone marrow aspirations and biopsies. We hope to achieve a sufficient sample size for epidemiological and clinical research on this unusual feature of lupus.

## Consent

Written informed consent was obtained from the patient for the publication of this report.
